# Integration of Deep Learning and Sequential Metabolism to Rapidly Screen Dipeptidyl Peptidase (DPP)-IV Inhibitors from *Gardenia jasminoides Ellis*

**DOI:** 10.3390/molecules28217381

**Published:** 2023-11-01

**Authors:** Huining Liu, Shuang Yu, Xueyan Li, Xinyu Wang, Dongying Qi, Fulu Pan, Xiaoyu Chai, Qianqian Wang, Yanli Pan, Lei Zhang, Yang Liu

**Affiliations:** 1 School of Chinese Materia Medica, Beijing University of Chinese Medicine, Beijing 102488, China; 17860505345@163.com (H.L.); yu13671036780@126.com (S.Y.); 15194178536@163.com (X.L.); sjztddxzxl1@163.com (X.W.); qidongying0327@163.com (D.Q.); 20210935110@bucm.edu.cn (F.P.); 20220935120@bucm.edu.cn (X.C.); wangqianqian041622@163.com (Q.W.); 2 Institute of Information on Traditional Chinese Medicine, China Academy of Chinese Medical Sciences, Beijing 100700, China; 3 Institute of Medical Innovation and Research, Peking University Third Hospital, Beijing 100191, China

**Keywords:** deep-learning model, sequential metabolism, DPP-IV inhibitor, *Gardenia jasminoides Ellis*, genipin 1-gentiobioside

## Abstract

Traditional Chinese medicine (TCM) possesses unique advantages in the management of blood glucose and lipids. However, there is still a significant gap in the exploration of its pharmacologically active components. Integrated strategies encompassing deep-learning prediction models and active validation based on absorbable ingredients can greatly improve the identification rate and screening efficiency in TCM. In this study, the affinity prediction of 11,549 compounds from the traditional Chinese medicine system’s pharmacology database (TCMSP) with dipeptidyl peptidase-IV (DPP-IV) based on a deep-learning model was firstly conducted. With the results, *Gardenia jasminoides Ellis* (GJE), a food medicine with homologous properties, was selected as a model drug. The absorbed components of GJE were subsequently identified through in vivo intestinal perfusion and oral administration. As a result, a total of 38 prototypical absorbed components of GJE were identified. These components were analyzed to determine their absorption patterns after intestinal, hepatic, and systemic metabolism. Virtual docking and DPP-IV enzyme activity experiments were further conducted to validate the inhibitory effects and potential binding sites of the common constituents of deep learning and sequential metabolism. The results showed a significant DPP-IV inhibitory activity (IC_50_ 53 ± 0.63 μg/mL) of the iridoid glycosides’ potent fractions, which is a novel finding. Genipin 1-gentiobioside was screened as a promising new DPP-IV inhibitor in GJE. These findings highlight the potential of this innovative approach for the rapid screening of active ingredients in TCM and provide insights into the molecular mechanisms underlying the anti-diabetic activity of GJE.

## 1. Introduction

Diabetes mellitus (DM) is a common metabolic disease characterized by hyperglycemia [1]. An epidemiological survey showed that the global prevalence of diabetes is increasing year by year, and it was predicted to rise to 10.2% (578 million) by 2030, with the vast majority of these cases being type II diabetes (T2D) [2]. Incretin-based therapy has recently surfaced as a viable treatment option for individuals with T2D [3]. The incretin system is composed of two hormones, namely, glucagon-like peptide-1 (GLP-1) and glucose-dependent insulinotropic polypeptide (GIP), which elicit the secretion of insulin from pancreatic β-cells in reaction to elevated levels of blood glucose [4]. Nonetheless, the instability of these peptides in vivo presents a significant obstacle due to their limited half-lives and vulnerability to degradation and inactivation by dipeptidyl peptidase (DPP)-IV enzymes [5]. To tackle this issue, a novel category of therapeutic agents for T2D called DPP-IV inhibitors are increasingly being utilized in clinical settings due to their advantageous physiological properties in reducing glucose levels. These include trogliptin, vildagliptin, and sitagliptin [6]. These agents function primarily by extending the degradation time of GLP-1 within the body, thereby promoting insulin secretion and inducing glucose concentration-dependent hypoglycemic effects [7]. Nevertheless, clinical investigations have demonstrated that the chemically synthesized DPP-IV inhibitors that are marketed are susceptible to adverse effects, such as headache, rash, diarrhea, and abnormal liver function [8,9]. Traditional Chinese medicine (TCM) is a class of drugs based on natural plants, many of which are homologous with medicine and food [10]. These drugs are gaining popularity because of several advantages: they often have fewer side effects and better patient tolerance, and they are relatively less expensive and are accepted due to a long history of use [11]. A variety of Chinese medicines, such as *Astragalus membranaceus*, *Mulberry leaves*, and *Radix scutellariae*, have demonstrated significant efficacy in lowering blood glucose levels both in vivo and in vitro [12,13,14,15]. TCMs can exert hypoglycemic effects through various mechanisms, such as inhibiting the activity of DPP-IV enzyme [16]. Therefore, screening for effective DPP-IV inhibitors with low side effects from TCMs is one of the important directions for the development of hypoglycemic drugs.

Drug discovery and development starts with target identification and ends with clinical trials [17]. Owing to the large number of assays and tests required and a high risk of failure, the whole process of developing a new drug generally takes 10–20 years as well as a capital investment, which ranges from USD 0.5 billion to USD 2.6 billion [18,19]. A major stage in the drug discovery process entails the identification of interactions between drugs and their respective targets, a task conventionally achieved through rigorous in vitro experiments. To mitigate the considerable expenditure of time and resources, there has been a growing emphasis on in silico methodologies [20]. Consequently, rather than embarking on an exhaustive in vitro exploration, the initial step involves virtual screening, followed by subsequent experimental validation of potential candidates. The emergence of drug–target interaction (DTI) based on artificial intelligence has become a crucial tool in drug discovery, and its progress has substantially improved the effectiveness of novel drug development [21]. DTI prediction serves as an important step in the process of drug discovery. More recently, deep-learning-based approaches have rapidly progressed for computational DTI prediction due to their successes in other areas, enabling large-scale validation in a relatively short time [22]. In this study, a deep-learning model, DrugBAN, was employed to identify the compound DPP-IV protein interactions. This is the state-of-the-art method in the prediction of compound–protein interactions and was reported to have excellent accuracy [23].

Contrary to the predominant “one target, one drug” approach of Western medicine, TCM is a multifaceted system that operates through the modulation of multiple physiological pathways, utilizing a variety of components and targets [24]. The complex chemical profile of TCM results in certain components being incapable of producing their intended effects. Only components that successfully reach the target and maintain an appropriate blood concentration are deemed therapeutically effective [25,26]. Therefore, targeting the absorbed ingredients by in vivo metabolic methods can effectively increase the hit rate of the active compound and simplify the description of the active substance [27]. Through this approach, Luo et al. introduced an integrated strategy founded upon a sequential metabolite identification approach, network pharmacology, molecular docking, and surface plasmon resonance (SPR) analysis. This method led to the successful identification of the active constituents within *Paeoniae Radix Alba* [28].

*Gardenia jasminoides Ellis* (GJE) is the dried and mature fruit of the Rubiaceae plant Gardenia and a kind of homologous food medicine that is rich in various bioactive compounds that exhibit a diverse range of pharmacological activities. Among them, iridoid glycosides and yellow pigment are generally considered the main bioactive and characteristic ingredients. GJE has a rich and wide range of cultivation resources, with a low price, which contributes to its low-cost characteristics. It is well known and frequently used not only as an excellent natural colorant, but also as an important traditional medicine for the treatment of different diseases, such as clearing away heat, cooling the blood, and eliminating stasis to activate blood circulation. It has demonstrated notable efficacy in anti-inflammatory, hypoglycemic, hepatoprotective, and cholagogic aspects and is widely used in Chinese clinical prescriptions [29]. Previous studies have reported that the 60% alcoholic extract of GJE exhibited significant hypoglycemic effects and improved insulin resistance [30,31]. Iridoid glycosides were identified as one of the major kinds of components in GJE and have demonstrated hypoglycemic effects in animal models [32]. However, the mechanism of action underlying these effects remains unclear. Furthermore, the absorption and metabolism of GJE and its primary active components also need to be investigated to evaluate their in vivo activity.

In this study, the interactions between 11,549 compounds from the traditional Chinese medicine systems pharmacology database (TCMSP) and DPP-IV were first predicted using a deep-learning model, and GJE was screened as a model drug for further study. Subsequently, sequence metabolism was employed to gain a more comprehensive understanding of the major absorbed components of GJE and their distribution in rats. This was followed by a comprehensive strategy of molecular docking and in vitro activity analyses to validate the potential active components in GJE. This approach combines deep-learning prediction, in vivo uptake distribution, and in vitro activity validation, and all experimental techniques and methods can be used as mature tools for screening and verifying related compounds. These tools do not depend on specific compounds, so they can be effectively extended to the study of more herbal ingredients. This approach holds great potential for similar studies in the future and can serve as a valuable methodological framework.

## 2. Results and Discussion

### 2.1. Validation of Deep-Learning Model

**Figure 1:**
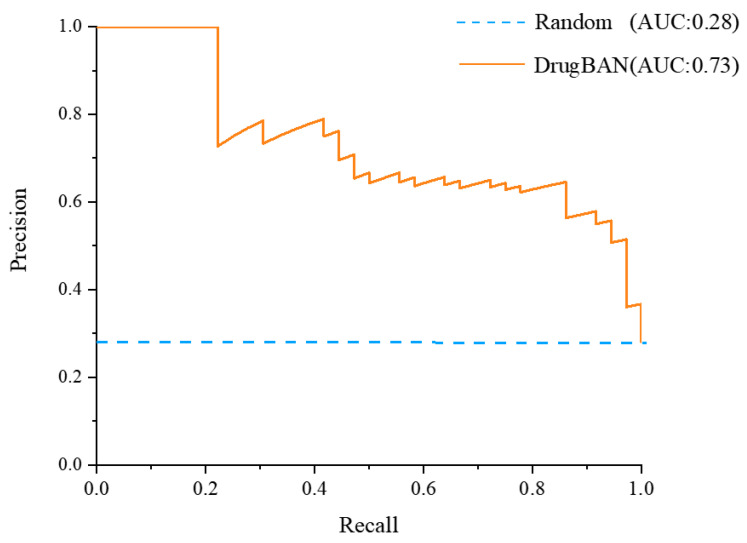
The true positive rate against the positive predictive value of DrugBAN and random prediction model.

The activities of compounds in TCM were predicted using the drugBAN model, with the code available at GitHub (https://github.com/peizhenbai/DrugBAN/tree/main, accessed on 1 June 2022). The DPP-IV dataset was collected from PubChem (https://pubchem.ncbi.nlm.nih.gov/, accessed on 1 June 2022) and used to train the model, which employed 80–20 splits for training and testing. Precision–recall curves plotting the true positive rate against the positive predictive value were then generated (Figure 1). It was observed that the area under the precision–recall curve (auPRC), which measures the ability of the model to correctly identify a compound, was favorable with a value of 0.73. This indicated that the model could more accurately identify DPP-IV-inhibitory compounds in our training set compared to random prediction (auPRC of 0.28). Further evaluation of the model’s performance using various metrics such as the AUROC, F1 score, sensitivity, specificity, accuracy, and threshold score also exhibited superior results in the DrugBAN model (Table 1).

### 2.2. Deep-Learning Model Prediction and Filters

The optimized model was applied to predict the DPP-IV-inhibitory activities of 11,549 compounds from the TCMSP Database (https://old.tcmsp-e.com/index.php, accessed on 2 June 2022). The interactions were predicted with scores ranging from 0 to 1. The prediction results for the 11,549 compounds interacting with DPP-IV are shown in Supplementary Table S1. The top 30 compounds with the best inhibition capacity were selected to identify their respective sources, and the results are presented in Table 2. We investigated the major botanical sources of the top 30 compounds and screened them on the basis of the frequency of their use in herbal medicines in clinical prescriptions, their level of toxicity (https://db.ouryao.com/, accessed on 2 June 2022), and their potential for food medicine with homologous properties (http://www.foodmate.net/, accessed on 2 June 2022). Although compounds such as genipin 1-gentiobioside and others did not exhibit particularly significant affinity, it is worth noting that a considerable portion of the top 30 hit compounds were derived from GJE. Therefore, GJE was chosen as the model drug for further investigation.

### 2.3. Identification of the Absorbed Components in Gardenia jasminoides Ellis

In order to provide a more comprehensive understanding of the major constituents of GJE and their distribution in rats, plasma samples obtained from different sites and different methods were analyzed using a sequential metabolism process [33], as reported previously. Briefly, the mesenteric vein/femoral vein plasma and the abdominal aorta plasma were collected from in situ intestinal perfusion and gavage, respectively. The mesenteric vein plasma samples were metabolized by enzymes of the intestinal wall, and both the intestine and liver contributed to the metabolic processes of the femoral vein plasma samples. Following metabolism by multiple organs and bacterial flora throughout the body, the abdominal aorta plasma samples served as a state of equilibrium. By comparing these samples, a clearer understanding of the absorption and metabolism site of GJE extract can be obtained. The extract of GJE was analyzed, and the results showed that a total of 46 components were identified, including iridoid glycosides, organic acids and their derivatives, monoterpenoids, and flavonoids, as illustrated in Supplementary Table S1. Moreover, a total of 38 prototype compounds were identified from drug-treated plasma samples through a meticulous comparison of their molecular formulas, fragment ions, and retention times with those of the parent compounds, with 38 in the mesenteric blood (MB) group, 25 in the femoral venous blood (FVB) group, and 14 in the abdominal aorta (AA) group, as illustrated in Table 3. DPP-IV is secreted by intestinal cells and enters the bloodstream to rapidly degrade GLP-1, thereby inhibiting its ability to stimulate insulin secretion [4,5]. Therefore, components absorbed in the mesenteric vein and systemic circulation were all included in the analyses. It is of significance to note that the predominant absorbed compounds in rat plasma were iridoid glycosides, which was consistent with the results predicted by the deep-learning model. Taken together, the above study could give a comprehensive map of the dynamic metabolic process of GJE, which would effectively narrow the range of potentially bioactive components of GJE.

### 2.4. Molecular Docking Studies

The compounds that could be predicted by deep-learning models and could also be absorbed into the bloodstream (e.g., genipin 1-gentiobioside, shanziside, geniposide, geniposidic acid, shanziside methyl ester, scandoside) were used for molecular docking to verify their interactions with the DPP-IV binding site to verify the credibility of the docking. The constructed conditional parameters were used to re-dock the sitagliptin. The parameters were judged to be reasonable based on whether the root mean square deviation (RMSD) was less than or equal to 2 Å [34]. The obtained RMSD value was 1.6079, indicating that the parameters were suitably set to reproduce the original binding pattern of the ligand receptor and were suitable for predicting the conformation of the ligand. The results indicated that the GJE components had been docked successfully with DPP-IV, as listed in Supplementary Table S2. Docking scores of the above six compounds absorbed into the blood and sitagliptin with DPP-IV are listed in the table below (Table 4).

**Figure 2:**
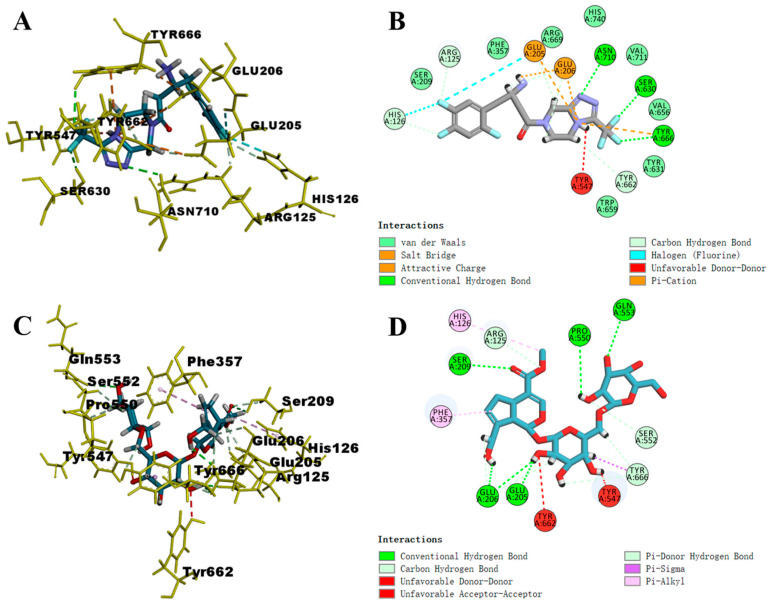
Molecular docking between sitagliptin, genipin 1-gentiobioside, and DPP-IV. (**A**) The specific residues of sitagliptin in the binding pockets/active sites of DPP-IV. (**B**) A 2D view of the interaction of sitagliptin with DPP-IV. (**C**) The specific residues of genipin 1-gentiobioside in the binding pockets/active sites of DPP-IV. (**D**) A 2D view of the interaction of genipin 1-gentiobioside with DPP-IV.

Understanding the molecular basis of ligand binding to receptors provides insights useful for rational drug design [35]. Based on Libdock scores, the docking of compounds with DPP-IV was analyzed using genipin 1-gentiobioside as an example. As a reference, sitagliptin and the three amino acid residues of the DPP-IV binding site each formed three hydrogen bonds (Figure 2B), and the C- and N-terminal residues established salt bridges with Glu205 and Glu206 (Figure 2A). Additionally, hydrophobic interactions were noted, which have been demonstrated to augment the inhibition of DPP-IV [36,37]. Regarding the docking mode of six compounds, it was found that, like sitagliptin, each compound could form various hydrogen bonds and hydrophobic interactions with key amino acid residues at the DPP-IV binding site. As shown in Figure 2D, the interaction between receptor and ligand occurred through hydrogen bond, hydrophobic, and electrostatic interactions. The molecule could establish five hydrogen bonds with DPP-IV, involving four oxygen atoms and one hydrogen atom from the glycosyl side chain. Additionally, it can engage with five residues located at the binding site of DPP-IV, specifically at positions Ser209, Glu205, Glu206, Pro550, and Gln553. The carboxymethyl and cyclopentane structures of the compound could form hydrophobic interactions with His126 and Phe357. As previously mentioned, they facilitated the substrate’s binding to the catalytic site of the enzyme. The docking pose of genipin 1-gentiobioside and 6 key residues are shown in Figure 2C.

**Figure 3:**
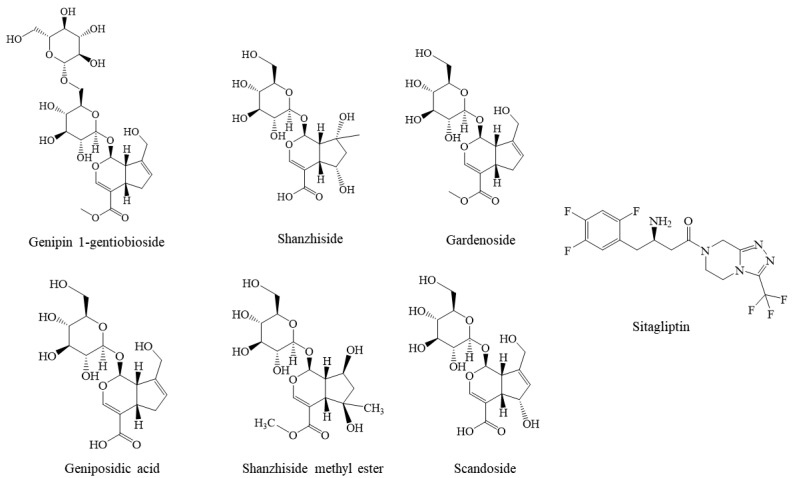
Molecular structures of six compounds with sitagliptin.

Overall, this integrated method combining UPLC-HRMS with computer analysis is effective for screening active ingredients from complex traditional Chinese medicine systems. Therefore, based on the virtual screening and docking process described above, combined with commercially available monomers, these six compounds were selected for subsequent DPP-IV inhibition experiments. The molecular structures of these six compounds and sitagliptin are shown in Figure 3.

### 2.5. In Vitro Activity Assay

**Figure 4:**
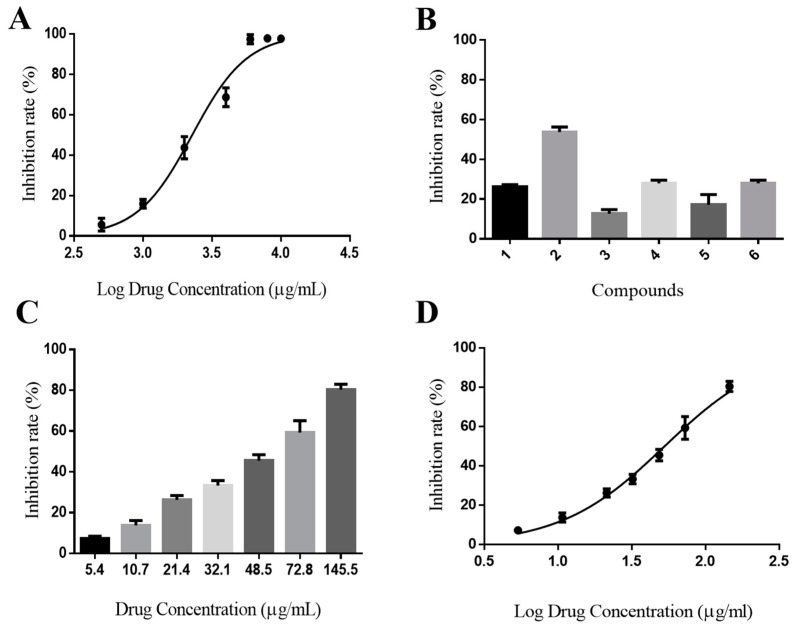
The DPP-IV inhibitory activity of *Gardenia jasminoides Ellis* extract, six iridoid glycosides, and iridoid glycosides. (**A**) The inhibition rate–concentration curve of GJE extracts. (**B**) Inhibitory activities of constituents 1–6 of GJE on DPP-IV in vitro. Final concentration of each sample: 1 mM. Data were expressed as mean ± SEM of triplicate experiments. (**C**) Concentration dependency inhibitory activity of the iridoid glycosides of GJE. (**D**) The inhibition rate–concentration curve of the iridoid glycosides.

The IC_50_ value for the positive control, sitagliptin, was 28.91 ± 0.33 nM, which was similar to that reported in the literature [38] and demonstrated the suitability of this system for activity determination. The present study identified that GJE showed certain inhibitory activity on DPP-IV (IC_50_ 2270 ± 230 μg/mL, Figure 4A). As shown in Figure 4C, the inhibitory activity showed a considerable concentration dependence manner. As shown in Figure 4B, the six GJE compounds inhibited the activity of DPP-IV, and genipin 1-gentiobioside (2) exhibited better anti-DPP-IV activity. The order of potency of the compounds tested was 2 > 6 > 4 > 1 > 5 > 3. This is consistent with the order of activity predicted by the DTI model. All compounds demonstrated concentration dependence. As the DPP-IV inhibitory activity demonstrated moderate potency, no activity assay at a higher concentration was conducted. The content of iridoid glycosides in GJE extract can be increased from 5.31% to 31.72% after D101 macroporous resin treatment, which indicates that this macroporous resin can be successfully used to enrich and purify iridoid glycosides in GJE, and it exhibits the most notable inhibitory activity (IC_50_ 53 ± 0.63 μg/mL) (Figure 4D). There is presumed to be a cooperative interaction between individual compounds on the inhibitory activity of DPP-IV.

This study found that the fraction of iridoid glycosides in GJE has significant inhibitory activity on DPP-IV. Genipin 1-gentiobioside has been identified as a novel and promising DPP-IV inhibitor, potentially serving as a lead compound for type 2 DM treatment. At the same time, extracting the active parts of natural products for the treatment of diabetes is also a promising strategy. However, it is important to note that the binding ability and selectivity of natural products towards the protein target require further enhancement when compared to commercially available drugs. To address this, future research should focus on designing and synthesizing a series of derivatives based on the ligand–receptor interaction mode results, aiming to improve the compounds’ affinity towards the target.

## 3. Materials and Methods

### 3.1. Materials

GJE was supplied by Beijing Tong Ren Tang Co., Ltd. (Beijing, China), which was identified by Prof. Jingjuan Wang (Beijing University of Chinese Medicine, Beijing, China). A voucher specimen has been deposited in B401 Laboratory of School of Chinese Materia Medica, Beijing University of Chinese Medicine (voucher No. 21122101). Methanol, MS-grade acetonitrile (purity ≥ 99.9%), and formic acid (purity ≥ 99) were provided by Thermo Fisher Scientific (Fairlawn, NJ, USA). Absolute ethanol (purity ≥ 99.9%) was supplied by Tianjin Damao Chemical Reagent Factory (Tianjin, China). Gardenoside, genipin, scandoside, genipin 1-gentiobioside, geniposidic acid, shanzhiside, shanzhiside methyl ester, and gardoside (purity ≥ 98%) were purchased from Nanjing Dilger Medical Technology Co., Ltd. (Nanjing, China). Recombinant human DPP-IV was provided by ProSpec (Rehovot, Israel). D101 adsorption resin was purchased from Tianjin Bailens Biotechnology Co., Ltd. (Tianjin, China).

### 3.2. Deep-Learning Model Predicts Compound Affinity for DPP-IV

#### 3.2.1. Data Collection and Preparation

The training dataset for this study was obtained from PubChem, comprising compounds that have been experimentally confirmed through assay. After representing them by SMILES, the collected compounds were curated to eliminate duplicates, inorganic material, and mixtures. Additionally, the protein sequences were extracted from the UniProt protein database using the UniProt ID as a reference. Accordingly, a dataset containing 1691 protein-drug samples was obtained, in which 536 were positive samples and 1155 were negative ones. A protein-drug sample is positive if the IC_50_ is less than 10 μM, or it is negative if the IC_50_ is greater than 10 μM. The data were divided into a training set (422 positive samples, 930 negative samples), a validation set (62 positive samples, 107 negative samples), and a test set (52 positive samples, 118 negative samples) with a guaranteed ratio of positive to negative samples.

#### 3.2.2. Deep-Learning Model

The deep-learning model used in this work builds on that applied in DrugBAN [35] (https://github.com/peizhenbai/DrugBAN/tree/main, accessed on 2 June 2022), a deep-learning bilinear attention network (BAN) framework with adversarial domain adaption to explicitly learn pair-wise interactions between drugs and targets. For each compound-protein pair, firstly, a graph-based molecular representation was generated from the compound’s simplified molecular-input line-entry system (SMILES) string, and a protein representation was encoded by 1D convolutional neural network (1D CNN) blocks from the protein sequence. Then, a bilinear attention network module was used to learn local interactions between encoded drug and protein representations. Finally, the interaction score was output by a fully connected classification layer. More detailed information is available in Ref. [39].

#### 3.2.3. Model Optimization and Evaluation

A binary activity value of 0 (no inhibition of DPP-IV activity) or 1 (possesses DPP-IV inhibitory activity) was assigned to each compound-protein pair in this work. To evaluate the model performance, we used the area under the receiver operating characteristic curve (AUROC) and the area under the precision–recall curve (AUPRC) as the major metrics. The training was performed for 100 epochs using random 80–10–10 training–validation–testing splits of the dataset. By default, and consistent with previous work [39], the binary cross entropy was used as the loss function. Precision–recall curves were generated by comparing the prediction score to the withheld activity value for each compound-protein pair in the testing subset using scikit-learn. In addition, we also report accuracy, sensitivity, and specificity at the threshold of the best F1 score (Supplementary Table S1).

#### 3.2.4. Model Prediction

For the final model, we used the full data (training, valid, and test data) to train. The model was then deployed to predict the compounds in HSD and the DPP-IV protein target score. There were several different source DPP-IV protein targets we selected to pair with each compound. Mouse, rat, and human DPP4 enzymes are very similar in structure, and the inhibitory effect of some inhibitors on the mouse enzyme may occur with the human enzyme as well. In the existing data study, some inhibitors used rat enzymes, some used mouse enzymes, and some used human enzymes to perform the experiment. These data were used as our training data to train the model. We wanted to make predictions for enzymes from different animals so as not to miss some compounds. The detailed information is shown in Table 5.

### 3.3. Preparation of Sample Solutions

The GJE was crushed into a fine powder using a grinder and subsequently subjected to sonication twice with 50% ethanol, at a solid–liquid ratio of 1:10 and at room temperature, for a duration of 20 min each time, using approximately 300 g of GJE powder. Then, the supernatant was transferred into an evaporating dish and concentrated using a water bath at 55 °C to 300 mL (1 g/mL), which was used for animal studies and macroporous resin column chromatography. The GJE solution (1 g/mL) was subjected to chemical analysis by diluting it to a concentration of 10 mg/mL crude drug. Six reference standards were individually dissolved in methanol using a 10 mL volumetric flask and stored at 4 °C. Prior to LC-MS analysis, the sample solution was filtered through a 0.22 µm pore size filter.

### 3.4. Enrichment of the Iridoid Glycoside Extract of GJE with Macroporous Resin

A D-101 macroporous resin column was used for the enrichment of the iridoid glycoside extract of GJE. Firstly, the effects of sample flow rate, sample concentration, elution solvent type, concentration, flow rate, and dosage on the adsorption efficiency were investigated separately. The enriched iridoid glycoside from the GJE extract was quantified by HPLC, using geniposide, genipin 1-gentiobioside, and geniposidic acid as the standard materials. The results showed that the best purification process, with a sample flow rate of 1 mL/min, a sample volume of 2.0 g of raw drug/g resin, an elution solvent of 30% ethanol, an elution flow rate of 2 mL/min, and a dosage of 30 mL, could effectively enrich and purify the iridoid glycosides of GJE.

### 3.5. Animals

Sprague–Dawley rats (males, 200–250 g) were procured from Spfanimals Laboratory Animal Technology Co., Ltd. (Beijing, China). The animals were maintained under controlled conditions of a 12 h light/dark cycle, a temperature range of 25–27 °C, and a relative humidity of 50–70%. Prior to the commencement of the study, the rats underwent an acclimatization period of no less than 7 days, during which they were provided ad libitum access to standard laboratory chow and water. Additionally, the animals were subjected to a 12 h water fast before the initiation of the experimental procedures. All protocols involving animal treatment in this study were ethically reviewed and approved by the Animal Ethics Committee of Beijing University of Chinese Medicine, under approval number BUCM-4-2022061502-2062.

### 3.6. Animal Experiments

#### 3.6.1. In Vivo Metabolic Experiments

**Figure 5:**
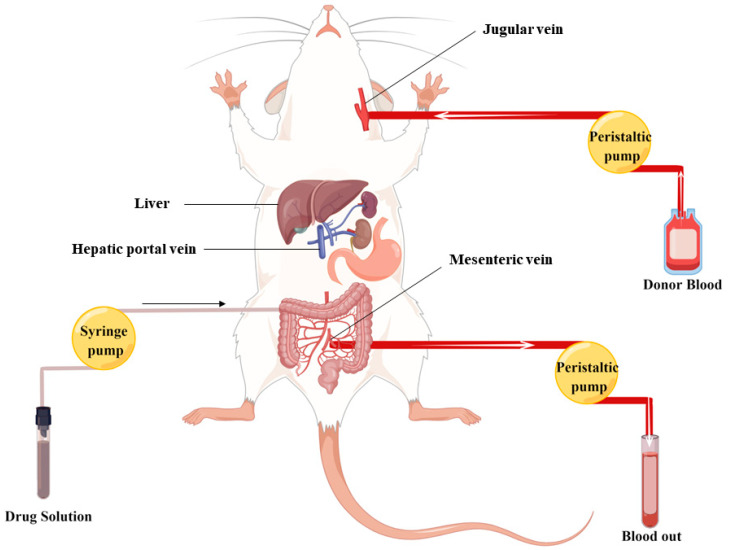
Schematic illustration depicting the process of in situ intestinal perfusion with venous sampling.

The in situ closed-loop technique is a well-established method utilized for the investigation of intestinal absorption. This approach enables the emulation of physiological conditions, allowing for the assessment of intestinal absorption over a predetermined timeframe. Notably, this model permits the precise measurement of absorption within specific anatomical segments of the rat intestine, namely, the jejunum, ileum, and colon [40]. The surgical procedures for IPVS were executed following established protocols outlined in the literature [41]. As shown in Figure 5, prior to the initiation of the perfusion surgery, five to seven rats were selected as blood donors for each experiment. Approximately 50–70 mL of blood was extracted from the abdominal aorta using a heparinized syringe and then incubated in a 37 °C water bath. This blood was subsequently prepared for transfusion into the recipient rat via the jugular vein, compensating for any blood loss through the mesenteric vein. The recipient rat was anesthetized through intraperitoneal administration of anesthetics, positioned supine on the operating table, and had its left external jugular vein exposed and cannulated with a 24-gauge i.v. catheter, facilitating the transfusion of blood from the donor reservoir. The abdominal cavity was meticulously opened along the abdominal line to expose the jejunum and the mesenteric/femoral veins. The jejunal segment ends were incised surgically, and two silicone tubes were carefully inserted and secured through a small incision. The jejunal segment was flushed with 37 °C saline until the effluent was clarified. Subsequently, the inlet tube was connected to the syringe pump. A catheter containing heparinized saline was then cannulated into the mesenteric vein (for intestinal wall metabolism) or femoral vein (for hepatic metabolism) and secured using instant glue. In cases involving the examination of intestinal wall absorption, the hepatic portal vein required ligation. The GJE solution (1 g/mL) was incubated in a water bath at 37 °C to maintain temperature. The solution was then pumped at a flow rate of 0.2 mL/min, and blood was pumped at a flow rate of 0.3 mL/min.

Within a span of two hours, mesenteric/femoral vein blood was collected into heparinized centrifuge tubes. Plasma was obtained through centrifugation of blood samples at 4000 rpm for 10 min, followed by protein precipitation using methanol. Following vortexing for 10 min, the mixture underwent centrifugation at 8000 rpm for an additional 10 min. The resulting organic layer was carefully transferred to a separate tube and subsequently dried under a stream of nitrogen at 40 °C. The resulting residue was then reconstituted in 200 µL of methanol for subsequent LC/MS analysis.

#### 3.6.2. Intragastric Administration

The rats were randomly divided into eight groups (three animals each). Subsequently, the treatment groups received a 4 mL oral gavage of GJE solution (1 g/mL), and the corresponding control groups were administered 4 mL of saline. Prior to experimentation, the rats underwent anesthesia via intraperitoneal injection of chloral hydrate (400 mg/kg). Subsequently, blood samples were collected from the abdominal aorta at specific time intervals (0.5, 1, 1.5, and 2 h), with three rats sampled at each time point. At the end of this study, all rats were sacrificed by conducting abilateral thoracotomy.

### 3.7. UPLC-Q Exactive-Orbitrap HRMS Analysis

Chromatographic separation was conducted using a Waters ACQUITY UPLC BEH Shield RP C18 column (100 mm × 2.1 mm, 1.7 µm) at a column temperature of 30 °C. The analytes were eluted through gradient elution using 0.1% formic acid in water (A) and acetonitrile (B) at a flow rate of 0.3 mL/min. A linear gradient of solvent B (*v*/*v*) was applied as follows: 0–1 min, 10% B; 1–4 min, 10–15% B; 4–18 min, 15–30% B; 18–24 min, 30–50% B; 24–28 min, 50–100% B; 28–31 min, 100% B; 31–32 min, 100–10% B; 32–35 min, 10% B. The injection volume was 5 µL.

The MS conditions were as follows: alternate switching (−)/(+) ESI full scan mode, the capillary temperature was 320 °C, auxiliary temperature was 250 °C, positive spray voltage was set at +3.5 kV, negative spray voltage was set at −3.0 kV, shealth gas (N_2_) flow was 35 Arb, aux gas flow rate was 10 Arb. Full MS scans were acquired in the range of *m*/*z* 100–1500, and the collision energy was set at 20, 30, and 40 eV. The MS/MS experiments were set as data-dependent scans. Data acquisition and processing were accomplished with Xcalibur software (version 4.2; Thermo Fisher Scientific).

### 3.8. Molecular Docking

The molecular docking process was conducted using the LibDock module of Discovery Studio 2019 software (Accelrys Software Inc., San Diego, CA, USA). The molecular structures of the compounds were obtained from the ChemSpider website (www.chemspider.com, accessed on 2 June 2022), and the crystal structure of DPP-IV, with the inhibitor vildagliptin bound in the active site, was obtained from the protein data bank (PDB ID:6B1E). Water molecules and co-crystallized ligands were removed from the protein structure. Atom types, charges, and hydrogen atoms were assigned to both the protein and ligand structures. A radius of 12 Å was set for the docking process, and the catalytic domain of DPP-IV consists of Ser630, Asp708, and His740 [42]. Also, Glu205 and Glu206 play a critical role in the activity of this enzyme [34,43]. Next, the 38 candidate ligand compounds were subjected to the “prepare ligands” module to match with the receptor. Subsequently, the ligands with diverse conformations were rigidly superimposed onto the map to ascertain the optimal interaction and energy optimization. The optimal conformation of each compound could be determined based on its highest docking score, followed by the arrangement of the compounds in descending order of their respective docking scores.

### 3.9. In Vitro DPP-IV Inhibition Assay

The DPP-IV inhibition assay was employed for in vitro biological activity evaluation of compounds [44]. Based on the results of DTI model prediction and the prototypical uptake of major components of GJE in rats, the iridoid glycosides’ potent fractions of GJE were enriched with macroporous resin, and the DPP-IV inhibitory activity of iridoid glycosides’ potent fractions and six iridoid glycosides, namely, geniposide (1), genipin 1-gentiobioside (2), scandoside (3), geniposidic acid (4), shanzhiside methyl ester (5), and shanzhiside (6), were tested (0.02, 0.05, 0.1, 0.2, 0.4, 0.8, and 1 mM). Sitagliptin was used as a positive control, and the assay was performed in 96-well microplates based on optimized conditions. Briefly, test compounds at various concentrations (40 µL), diluted with assay buffer (0.1 M Tris-HCl buffer, 0.1 M NaCl, and 1 mM EDTA, pH = 8.0), were added to a 96-well clear-bottom microtiter plate. Subsequently, 20 µL of 1.75 mM Gly-Pro-pNA was added and thoroughly mixed, followed by a 10 min incubation at 37 °C. The reaction was initiated by adding 40 µL of 0.4 µg/mL human recombinant DPP-IV. A control group without the inhibitor was also included. After a 30 min incubation, absorbance was measured at 405 nm using a Skanlt RE absorbance reader (Thermo Scientific, San Jose, CA, USA), and IC_50_ values were calculated using the provided equation:

$$\mathrm{DPP}\text{-}\mathrm{IV}\;\mathrm{inhibition}(\%)=\left[1-\frac{A\left(sample\right)-A(sample\;control)}{A\left(negative\;reaction\right)-A(negative\;control)}\right]\times 100\%$$

In the provided experimental setup: A(sample control) included both the sample and substrate (Gly-Pro-pNA). A(negative reaction) comprised DPP-IV and substrate. A(negative control) solely contained the substrate. The term IC_50_ denotes the concentration of inhibitors necessary to suppress 50% of DPP-IV activity.

## 4. Conclusions

This study presents a novel approach containing deep-learning model prediction, absorption and metabolism characteristics, virtual screening, and target activity screening. This powerful approach can be effectively employed for the discovery of potential active ingredients or lead compounds in TCM. This finding presents a novel avenue for the exploration of potent and low-toxicity DPP-IV inhibitors derived from herbal medicine. This study found that the effective fraction of iridoid glycosides in GJE has significant inhibitory activity on DPP-IV, which will guide the effective development of GJE as a hypoglycemic Chinese medicine in the future. In addition, it can also be used as a dietary supplement for the prevention and adjuvant treatment of diabetes. In contrast, the compound genipin 1-gentiobioside has been identified as a promising novel DPP-IV inhibitor that is effective, low-cost, and non-toxic, and its potential as a lead compound for the management of T2D warrants further investigation. In our research, we utilized the DrugBAN model [39] to swiftly forecast the activity of over 10,000 traditional Chinese medicine compounds in a dataset. This computational methodology yielded outcomes within a brief one-week period. Conversely, conventional experimental techniques not only involve significant financial costs but also necessitate several months to obtain the corresponding activity data. These findings demonstrate that combining experiment with computation and deep learning can enable one to efficiently discover DPP-IV inhibitory compounds in TCM and rapidly elucidate their potential mechanisms of action. Despite the promising nature of our deep-learning approach, it is imperative to acknowledge its inherent limitations. Deep-learning techniques are commonly restricted by the quality and diversity of the utilized training data. Although our dataset comprised 1691 compound-protein samples, providing an adequate number of active compounds for training models with predictive capabilities, enhancing the structural diversity within the training set is imperative. This augmentation will enable models to better discern the chemical substructures responsible for conferring activity, thereby facilitating the discovery of a wider array of structurally diverse inhibitors. Overall, this innovative method has great potential for the rapid screening of active ingredients in TCM and provides a new research idea and material basis for the development of new T2D drugs.

## Figures and Tables

**Table 1:** Model performance comparison between trained and random prediction model.

Metrics	Trained Model	Random Prediction
AUROC	0.8810	0.5000
AUPRC	0.7319	0.2813
F1 score	0.9729	-
Sensitivity	0.9865	-
Specificity	0.9600	-
Accuracy	0.9832	-
Threshold	0.2706	-

**Table 2:** Top 30 compounds’ sources and predicted affinity scores with different sources of DPP-IV.

No.	Source of Compound	Compound’s Name	TCMSP ID	P27487 Score	P28843 Score	P14740 Score	Total Score
**1**	*Ecliptae Herba*	Ecliptasaponin D	MOL003383	0.9798	0.8247	0.8418	2.6463
**2**	*Xanthium sibiricum Patr.*	Strumaroside	MOL002294	0.9796	0.8506	0.9227	2.7529
**3**	*Ephedra Herba*	2,6-Dimethyl-1,3,5,7-octatetraene, *E*,*E*-	MOL003935	0.9795	0.7253	0.8179	2.5227
**4**	*Magnolia Officinalis Rehd Et Wils.*	5-allyl-3-(4-allylphenoxy)pyrocatechol	MOL005972	0.9795	0.6784	0.8428	2.5007
**5**	*Myrrha*	3β-acetoxy-16β-hydroxydammar-24-ene	MOL001150	0.9793	0.9315	0.9425	2.8533
**6**	*Dictamni Cortex*	Dictamnoside	MOL006254	0.9793	0.7955	0.8364	2.6112
**7**	*Radix Paeoniae Rubra*	1-*O*-β-d-glucopyranosyl-8-*O*-benzoylpaeonisuffrone	MOL006993	0.9793	0.6134	0.7151	2.3078
**8**	*Tribulifructus*	Spirostanol	MOL008580	0.9792	0.7898	0.9191	2.6881
**9**	*Gardenia jasminoides Ellis*	Genipin 1-gentiobioside	MOL009038	0.9792	0.7189	0.7703	2.4684
**10**	*Dysosmae Verspiellis Rhixoma Et Radix*	(E)-4-[(1S)-2,6,6-trimethyl-1-cyclohex-2-enyl]but-3-en-2-one	MOL011707	0.9791	0.8813	0.886	2.7464
**11**	*A. Dahurica (Fisch.) Benth. Et Hook*	Daturic acid	MOL011501	0.9788	0.844	0.8818	2.7046
**12**	*Polygonati Rhizoma*	Sibiricoside B	MOL009762	0.9787	0.8104	0.8806	2.6697
**13**	*Gardenia jasminoides Ellis*	Shanziside	MOL004560	0.9786	0.8053	0.8381	2.622
**14**	*Croci Stigma*	Carthamin	MOL001413	0.9786	0.6905	0.8393	2.5084
**15**	*Ginkgo Semen*	Amentoflavone	MOL012037	0.9786	0.9096	0.9011	2.7893
**16**	*Pulsatilliae Radix*	5,6,7-Trimethoxycoumarin	MOL011997	0.9786	0.9096	0.9011	2.7893
**17**	*Arum Ternatum Thunb.*	(Z)-3-(4-hydroxy-3-methoxyphenyl)prop-2-enoic acid	MOL010389	0.9784	0.8322	0.9062	2.7168
**18**	*Gardenia jasminoides Ellis*	Gardenoside	MOL004554	0.9784	0.8015	0.7198	2.4997
**19**	*Hedysarum Multijugum Maxim.*	Isoflavanone	MOL010398	0.9784	0.8322	0.9062	2.7168
**20**	*Imperatae Rhizoma*	Bifendate	MOL010387	0.9784	0.8322	0.9062	2.7168
**21**	*Gardenia jasminoides Ellis*	Geniposidic acid	MOL001668	0.9782	0.8552	0.8563	2.6897
**22**	*Hedysarum Multijugum Maxim.*	AstragalosideⅢ_	MOL010406	0.9782	0.8322	0.9062	2.7166
**23**	*Carthami Flos*	Carthamin-precursor	MOL002779	0.9782	0.6842	0.838	2.5004
**24**	*Impatientis Semen*	Hosenkosides C	MOL008609	0.9782	0.8973	0.9492	2.8247
**25**	*Isatidis Radix*	5-(methoxymethyl)-2-furoic acid	MOL011822	0.9782	0.8899	0.9447	2.8128
**26**	*Gardenia jasminoides Ellis*	1,8-dihydroxy-3-Methylol-9,10-anthraquinone	MOL010471	0.9776	0.6499	0.7134	2.3409
**27**	*Radix Cynanchi Paniculati*	Tomentogenin	MOL005622	0.9774	0.9162	0.9443	2.8379
**28**	*Isatidis Radix*	3-[2′-(5′-hydroxymethyl)furyl]-1(2H)-isoquinolinone-7-*O*-β-d-glucoside	MOL011727	0.9774	0.8676	0.9538	2.7988
**29**	*Gardenia jasminoides Ellis*	Scandoside	MOL003135	0.9780	0.8923	0.8477	2.718
**30**	*Eupatorium Fortunei Turcz*	Taraxasteryl palmitate	MOL000605	0.9780	0.8676	0.9538	2.7994

**Table 3:** Identification of compounds from *Gardenia jasminoides Ellis* and the absorbed components in different plasma samples.

Peak No.	t_R_/min	Measured Mass	Error (ppm)	Molecular Formula	Prototypical Compounds	MB	FVB	AA
**1**	0.91	[M − H]^−^ 191.055 9	1.555	C_7_H_12_O_6_	Quinic acid	+	+	+
**2**	0.98	[M − H]^−^ 173.045 3	1.512	C_7_H_10_O_5_	Shikimic acid	-	-	-
**3**	2.65	[M − H]^−^ 391.124 5	1.134	C_16_H_24_O_11_	Shanzhiside isomers	+	+	+
**4**	2.81	[M − H]^−^ 403.124 1	0.183	C_17_H_24_O_11_	Deacetylasperulosidic acid methyl ester	-	-	-
**5**	2.84	[M − H]^−^ 389.108 7	0.883	C_16_H_22_O_11_	Scandoside	+	+	-
**6**	2.98	[M − H]^−^ 373.113 7	0.639	C_16_H_22_O_10_	Gardoside *	+	+	-
**7**	3.13	[M − H]^−^ 391.124 5	1.221	C_16_H_24_O_11_	Shanziside *	+	+	+
**8**	3.32	[M − H]^−^ 403.124 1	1.101	C_17_H_24_O_11_	Gardenoside	+	+	+
**9**	3.35	[M − H]^−^ 373.113 6	0.478	C_16_H_22_O_10_	Geniposidic acid *	+	+	+
**10**	3.38	[M − H]^−^ 403.124 1	−0.561	C_17_H_24_O_11_	Feretoside	+	+	+
**11**	3.48	[M − H]^−^ 405.139 9	0.503	C_17_H_26_O_11_	Shanziside methyl ester *	+	+	+
**12**	3.57	[M − H]^−^ 375.129 4	0.715	C_16_H_24_O_10_	Mussaenosidic acid	+	+	+
**13**	3.73	[M − H]^−^ 345.155 2	0.775	C_16_H_26_O_8_	Jasminoside D	+	+	+
**14**	3.96	[M − H]^−^ 327.144 7	0.832	C_16_H_24_O_7_	Zataroside B	+	+	+
**15**	4.04	[M − H]^−^ 353.087 6	1.028	C_16_H_18_O_9_	5/3-O-Caffeoyl-quinic acid	+	+	-
**16**	4.2	[M − H]^−^ 549.181 5	−0.883	C_23_H_34_O_15_	Genipin 1-gentiobioside	-	-	+
**17**	4.87	[M − H]^−^ 387.129 4	0.693	C_17_H_24_O_10_	Geniposide *	+	+	+
**18**	5.34	[M − H]^−^ 345.155 2	0.775	C_16_H_26_O_8_	Jasminoside B	+	+	-
**19**	5.49	[M − H]^−^ 353.087 4	0.405	C_16_H_18_O_9_	Chlorogenic acid	+	+	-
**20**	6.33	[M − H]^−^ 179.034 8	1.832	C_9_H_8_O_4_	Caffeic acid	+	+	-
**21**	6.39	[M − H]^−^ 183.102 3	1.206	C_10_H_16_O_3_	Jasminodiol	+	+	+
**22**	6.62	[M − H]^−^ 503.176 9	0.883	C_22_H_32_O_13_	2-methyl-lerythritol-4-*O*-(6-*O*-transsinapoyl)-β-d-glucopyranoside	+	+	-
**23**	7.01	[M − H]^−^ 359.134 7	1.372	C_16_H_24_O_9_	Ixoroside	+	+	+
**24**	7.44	[M − H]^−^ 429.139 8	0.335	C_19_H_26_O_11_	10-acetyl geniposide	+	+	-
**25**	7.57	[M − H]^−^ 519.150 6	0.614	C_25_H_28_O_12_	6′-*O*-trans-coumaroyl geniposidic acid	+	-	-
**26**	8.24	[M − H]^−^ 551.176 8	0.643	C_26_H_32_O_13_	6-*O*-trans-p-coumaroyl Gardenoside methyl ester	+	-	-
**27**	9.89	[M − H]^−^ 491.213 3	0.954	C_22_H_36_O_12_	Jasminoside S/H/I	+	-	-
**28**	10.16	[M − H]^−^ 579.172 1	1.174	C_27_H_32_O_14_	6′-*O*-trans-sinapoyl gardoside	+	-	-
**29**	10.43	[M − H]^−^ 565.192 4	0.574	C_27_H_34_O_13_	11-(6-*O*-trans-sinapoylglucopyranosyl)gardendiol	+	-	-
**30**	10.79	[M − H]^−^ 609.146 3	1.216	C_27_H_30_O_16_	Rutin	-	-	-
**31**	11.6	[M − H]^−^ 465.101 8	0.938	C_21_H_20_O_12_	Isoquercitrin	-	-	-
**32**	11.86	[M − H]^−^ 593.151 3	1.121	C_27_H_30_O_15_	Nicotiflorin	+	-	-
**33**	12.12	[M − H]^−^ 755.240 8	1.306	C_34_H_44_O_19_	6″-*O*-trans-sinapoylgenipin gentiobioside	+	-	-
**34**	12.62	[M − H]^−^ 725.230 2	1.201	C_33_H_42_O_18_	6″-*O*-trans-feruloyl genipin gentiobioside	+	-	-
**35**	12.69	[M − H]^−^ 695.219 2	0.742	C_32_H_40_O_17_	6″-*O*-trans-p-coumaroylge nipin gentiobioside	+	-	-
**36**	13.08	[M − H]^−^ 551.213 3	0.742	C_27_H_36_O_12_	6′-*O*-trans-sinapoyl Jasminoside L	+	-	-
**37**	13.34	[M − H]^−^ 975.371 0	0.044	C_44_H_64_O_24_	trans-crocin Ⅰ/cis-crocin Ⅰ	-	-	-
**38**	14.07	[M − H]^−^ 593.187 7	1.095	C_28_H_34_O_14_	6′-*O*-sinapoylgeniposide	+	-	-
**39**	14.64	[M − H]^−^ 515.119 1	0.251	C_25_H_24_O_12_	3,5-Dicaffeoylquinic acid	+	+	-
**40**	15.51	[M − H]^−^ 533.166 3	0.786	C_26_H_30_O_12_	6′-*O*-p-coumaroylgeniposide	+	+	-
**41**	15.67	[M − H]^−^ 659.162 1	1.366	C_31_H_32_O_16_	3,4-dicaffeovl-5-(3-hydroxy-3-methyl glutaroyl) quinic acid	+	+	-
**42**	16.44	[M − H]^−^ 559.145 5	0.616	C_27_H_28_O_13_	3-caffeoyl-4-sinapoylquinate	+	+	-
**43**	17.45	[M − H]^−^ 535.218 3	0.716	C_27_H_36_O_11_	6′-*O*-trans-sinapoyl jasminoside A	-	-	-
**44**	18.57	[M − H]^−^ 533.202 5	0.419	C_27_H_34_O_11_	6′-*O*-trans-sinapoyl jasminoside C	+	-	-
**45**	19.58	[M − H]^−^ 345.061 4	0.95	C_17_H_14_O_8_	5,7,3′,4′-tetrahydroxy-6,8-dimethoxy flavone	+	-	-
**46**	21.75	[M − H]− 813.319 2	1.286	C_38_H_54_O_19_	Crocin II	-	-	-

Note: MB: mesenteric blood; FVB: femoral venous blood; AA: abdominal aorta. * means components were compared with reference standards. + means that the component is absorbed into the blood. - means that the component is not absorbed into the blood.

**Table 4:** LibDock scores for 6 prototype compounds and sitagliptin.

Num	Name	Libdock Score
**1**	Genipin 1-gentiobioside	142.425
**2**	Shanzhiside	142.425
**3**	Sitagliptin	136.846
**4**	Gardenoside	132.894
**5**	Geniposidic acid	127.404
**6**	Shanzhiside methyl ester	107.752
**7**	Scandoside	107.136

**Table 5:** Selected DPP-IV proteins information.

UniProt ID	Protein	Organism
P27487	Dipeptidyl peptidase 4	Homo sapiens (human)
P28843	Dipeptidyl peptidase 4	Mus musculus (mouse)
P14740	Dipeptidyl peptidase 4	Rattus norvegicus (rat)

## Data Availability

All data included in this study are available upon request by contact with the corresponding author.

## Supplementary Materials

The following supporting information can be downloaded at: https://www.mdpi.com/article/10.3390/molecules28217381/s1.

## References

[1] Putta S., Yarla N.S., Kilari E.K., Surekha C., Aliev G., Divakara M.B., Santosh M.S., Ramu R., Zameer F., Prasad M.N.N. (2016). Therapeutic Potentials of Triterpenes in Diabetes and its Associated Complications. Curr. Top. Med. Chem..

[2] Samandari R., Chizari A., Hassanpour R., Mousavi Z., Haghparast A. (2013). Streptozotocin-induced diabetes affects the development morphine reward in rats. Neurosci. Lett..

[3] Vorsanger M.H., Subramanyam P., Weintraub H.S., Lamm S.H., Underberg J.A., Gianos E., Goldberg I.J., Schwartzbard A.Z. (2016). Cardiovascular Effects of the New Weight Loss Agents. J. Am. Coll. Cardiol..

[4] Nauck M. (2016). Incretin therapies: Highlighting common features and differences in the modes of action of glucagon-like peptide-1 receptor agonists and dipeptidyl peptidase-4 inhibitors. Diabetes Obes. Metab..

[5] Kerr B.D., Flatt P.R., Gault V.A. (2010). Effects of gamma-glutamyl linker on DPP-IV resistance, duration of action and biological efficacy of acylated glucagon-like peptide-1. Biochem. Pharmacol..

[6] Sharma M.D. (2015). Potential for combination of dipeptidyl peptidase-4 inhibitors and sodium-glucose co-transporter-2 inhibitors for the treatment of type 2 diabetes. Diabetes Obes. Metab..

[7] Singh A.K., Yadav D., Sharma N., Jin J.O. (2021). Dipeptidyl Peptidase (DPP)-IV Inhibitors with Antioxidant Potential Isolated from Natural Sources: A Novel Approach for the Management of Diabetes. Pharmaceuticals.

[8] Li C.J., Liu X.J., Bai L., Yu Q., Zhang Q.M., Yu P., Yu D.M. (2014). Efficacy and safety of vildagliptin, Saxagliptin or Sitagliptin as add-on therapy in Chinese patients with type 2 diabetes inadequately controlled with dual combination of traditional oral hypoglycemic agents. Diabetol. Metab. Syndr..

[9] Howse P.M., Chibrikova L.N., Twells L.K., Barrett B.J., Gamble J.M. (2016). Safety and Efficacy of Incretin-Based Therapies in Patients With Type 2 Diabetes Mellitus and CKD: A Systematic Review and Meta-analysis. Am. J. Kidney Dis..

[10] Shu Y.S., He D., Li W., Wang M.L., Zhao S.Y., Liu L.L., Cao Z.W., Liu R., Huang Y.J., Li H. (2020). Hepatoprotective Effect of Citrus aurantium L. Against APAP-induced Liver Injury by Regulating Liver Lipid Metabolism and Apoptosis. Int. J. Biol. Sci..

[11] Li L.S., Zhang J.L., Qiao Q.H., Wu L.H., Chen L.Y. (2020). Development, Reliability, and Validity of the “Knowledge-Attitude-Practice” Questionnaire of Foreigners on Traditional Chinese Medicine Treatment. Evid.-Based Complement. Altern. Med..

[12] Chen X.X., Chen C., Fu X. (2022). Hypoglycemic activity in vitro and vivo of a water-soluble polysaccharide from Astragalus membranaceus. Food Funct..

[13] Li J.S., Ji T., Su S.L., Zhu Y., Chen X.L., Shang E.X., Guo S., Qian D.W., Duan J.A. (2022). Mulberry leaves ameliorate diabetes via regulating metabolic profiling and AGEs/RAGE and p38 MAPK/NF-kappa B pathway. J. Ethnopharmacol..

[14] Yan X.M., Zhang Y.L., Peng Y., Li X.B. (2022). The water extract of Radix scutellariae, its total flavonoids and baicalin inhibited CYP7A1 expression, improved bile acid, and glycolipid metabolism in T2DM mice. J. Ethnopharmacol..

[15] Shi J.N., Hu H., Harnett J., Zheng X.T., Liang Z.J., Wang Y.T., Ung C.O.L. (2019). An evaluation of randomized controlled trials on nutraceuticals containing traditional Chinese medicines for diabetes management: A systematic review. Chin. Med..

[16] Ma H.H., Zhang J., Li C.Q., Zou L.W. (2023). Discovery of anthraquinones as DPP-IV inhibitors: Structure-activity relationships and inhibitory mechanism. Fitoterapia.

[17] Rifaioglu A.S., Atas H., Martin M.J., Cetin-Atalay R., Atalay V., Dogan T. (2019). Recent applications of deep learning and machine intelligence on in silico drug discovery: Methods, tools and databases. Brief. Bioinform..

[18] Xue H., Li J., Xie H., Wang Y. (2018). Review of Drug Repositioning Approaches and Resources. Int. J. Biol. Sci..

[19] Cheuka P.M., Mayoka G., Mutai P., Chibale K. (2017). The Role of Natural Products in Drug Discovery and Development against Neglected Tropical Diseases. Molecules.

[20] Peska L., Buza K., Koller J. (2017). Drug-target interaction prediction: A Bayesian ranking approach. Comput. Methods Programs Biomed..

[21] Ezzat A., Wu M., Li X.L., Kwoh C.K. (2019). Computational prediction of drug-target interactions using chemogenomic approaches: An empirical survey. Brief. Bioinform..

[22] Perez-Lopez C., Molina A., Lozoya E., Segarra V., Municoy M., Guallar V. (2023). Combining machine-learning and molecular-modeling methods for drug-target affinity predictions. Wiley Interdiscip. Rev.-Comput. Mol. Sci..

[23] Bagherian M., Sabeti E., Wang K., Sartor M.A., Nikolovska-Coleska Z., Najarian K. (2021). Machine learning approaches and databases for prediction of drug-target interaction: A survey paper. Brief. Bioinform..

[24] Li J.J., Li L., Wang Y.Y., Zhao Y.X., Hu P., Xu Z., Liu F., Liang Q.Q., Tian X.T., Huang C.G. (2021). Systematic investigation on the anti-rheumatoid arthritis material basis and mechanism of Juan Bi Tang. Part 1: Integrating metabolic profiles and network pharmacology. J. Pharm. Biomed. Anal..

[25] Zhang P.L., He S.R., Wu S.Q., Li Y., Wang H.Y., Yan C.Y., Yang H., Li P. (2022). Discovering a Multi-Component Combination against Vascular Dementia from Danshen-Honghua Herbal Pair by Spectrum-Effect Relationship Analysis. Pharmaceuticals.

[26] Tian X.T., Xu Z., Chen M.C., Hu P., Liu F., Sun Z.L., Liu H., Guo X.Z., Li Z.X., Huang C.G. (2018). Simultaneous determination of eight bioactive compounds by LC-MS/MS and its application to the pharmacokinetics, liver first-pass effect, liver and brain distribution of orally administrated Gouteng-Baitouweng (GB) in rats. J. Chromatogr. B-Anal. Technol. Biomed. Life Sci..

[27] Liao M.L., Shang H.H., Li Y.Z., Li T., Wang M., Zheng Y.A., Hou W.B., Liu C.X. (2018). An integrated approach to uncover quality marker underlying the effects of Alisma orientale on lipid metabolism, using chemical analysis and network pharmacology. Phytomedicine.

[28] Luo Z.Q., Liu Y., Han X., Yang W.N., Wang G.P., Wang J., Jiang X.Q., Sen M.L., Li X.Y., Yu G.H. (2021). Mechanism of Paeoniae Radix Alba in the Treatment of Non-alcoholic Fatty Liver Disease Based on Sequential Metabolites Identification Approach, Network Pharmacology, and Binding Affinity Measurement. Front. Nutr..

[29] Zhou H.B., Zhang S., Chen L.H., Liu Y.M., Shen L.H., Zhang J.L. (2023). Effective Therapeutic Verification of Crocin I, Geniposide, and Gardenia (*Gardenia jasminoides Ellis*) on Type 2 Diabetes Mellitus In Vivo and In Vitro. Foods.

[30] Ye Z.Y., Chen X., He Y.L., Jin M.Z., Ye M. (2021). Antidiabetic effects of fermented milk contained with Gardenia jasminoides water extracts on streptozotocin-induced mice. J. Food Process. Preserv..

[31] Guan L.L., Feng H.Y., Gong D.Z., Zhao X., Cai L., Wu Q., Yuan B., Yang M., Zhao J., Zou Y. (2013). Genipin ameliorates age-related insulin resistance through inhibiting hepatic oxidative stress and mitochondrial dysfunction. Exp. Gerontol..

[32] Miura T., Nishiyama Y., Ichimaru M., Moriyasu M., Kato A. (1996). Hypoglycemic activity and structure-activity relationship of iridoidal glycosides. Biol. Pharm. Bull..

[33] Luo Z.Q., Ma X.Y., Liu Y., Lu L.N., Yang R.R., Yu G.H., Sun M.H., Xin S.K., Tian S.M., Chen X.J. (2016). An Approach to Characterizing the Complicated Sequential Metabolism of Salidroside in Rats. Molecules.

[34] Qiao Y., Deng H., Liu L., Liu S., Ren L., Shi C., Chen X., Guan L., Liu W., Li Z. (2023). Highly Accessible Computational Prediction and In Vivo/In Vitro Experimental Validation: Novel Synthetic Phenyl Ketone Derivatives as Promising Agents against NAFLD via Modulating Oxidoreductase Activity. Oxid. Med. Cell. Longev..

[35] Dong M., Vattelana A.M., Lam P.C.H., Orry A.J., Abagyan R., Christopoulos A., Sexton P.M., Haines D.R., Miller L.J. (2015). Development of a Highly Selective Allosteric Antagonist Radioligand for the Type 1 Cholecystokinin Receptor and Elucidation of Its Molecular Basis of Binding. Mol. Pharmacol..

[36] Zhao L., Zhang M., Pan F., Li J., Dou R., Wang X., Wang Y., He Y., Wang S., Cai S. (2021). analysis of novel dipeptidyl peptidase-IV inhibitory peptides released from antimicrobial protein 2 (MiAMP2) and the possible pathways involved in diabetes protection. Curr. Res. Food Sci..

[37] Araki M., Kanegawa N., Iwata H., Sagae Y., Ito K., Masuda K., Okuno Y. (2020). Hydrophobic interactions at subsite S1′ of human dipeptidyl peptidase IV contribute significantly to the inhibitory effect of tripeptides. Heliyon.

[38] Kang B., Skonberg D.I., Myracle A.D. (2020). Anti-Hyperglycemic Effects of Green Crab Hydrolysates Derived by Commercially Available Enzymes. Foods.

[39] Bai P., Miljković F., John B., Lu H. (2023). Interpretable bilinear attention network with domain adaptation improves drug-target prediction. Nat. Mach. Intell..

[40] Luo Z.Q., Liu Y., Zhao B.S., Tang M.M., Dong H.H., Zhang L., Lv B.R., Wei L. (2013). Ex vivo and in situ approaches used to study intestinal absorption. J. Pharmacol. Toxicol. Methods.

[41] Li H.W., Dong L., Liu Y., Wang G., Zhang L., Qiao Y.J. (2014). Comparison of two approaches of intestinal absorption by puerarin. J. Pharmacol. Toxicol. Methods.

[42] Patel B.D., Ghate M.D. (2014). Recent approaches to medicinal chemistry and therapeutic potential of dipeptidyl peptidase-4 (DPP-4) inhibitors. Eur. J. Med. Chem..

[43] Musoev A., Numonov S., You Z.H., Gao H.W. (2019). Discovery of Novel DPP-IV Inhibitors as Potential Candidates for the Treatment of Type 2 Diabetes Mellitus Predicted by 3D QSAR Pharmacophore Models, Molecular Docking and De Novo Evolution. Molecules.

[44] Sharma R., Soman S.S. (2015). Design and synthesis of sulfonamide derivatives of pyrrolidine and piperidine as anti-diabetic agents. Eur. J. Med. Chem..

