# Supplementary material for: Integration of Deep Learning and Sequential Metabolism to Rapidly Screen Dipeptidyl Peptidase (DPP)-IV Inhibitors from Gardenia jasminoides Ellis
Source: Molecules. 2023 Nov 1;28(21):7381. doi: 10.3390/molecules28217381 (PMC10649927; doi:10.3390/molecules28217381)
Supplement: Supplementary file 1 [file molecules-28-07381-s001.zip › Supplementary Table S1.pdf]

peak No.	t_R/min	Ionisation mode	Mass(m/z)		Error (ppm)	Molecular formula	MS/MS fragments	Identification compound
			Theoretical Mass	Measured Mass				
1	0.91	$[M-H]^-$	191.056 1	191.055 9	1.555	$C_7H_{12}O_6$	191.056 1	Quinic acid
2	0.98	$[M-H]^-$	173.045 5	173.045 3	1.512	$C_7H_{10}O_5$	137.024 6, 111.045 4, 93.034 7	Shikimic acid
3	2.65	$[M-H]^-$	391.124 6	391.124 5	1.134	$C_{16}H_{24}O_{11}$	229.072 1, 167.071 3	Shanzhiside Isomers
4	2.81	$[M-H]^-$	403.124 6	403.124 1	0.183	$C_{17}H_{24}O_{11}$	241.072 3, 223.061 2, 127.040 2, 101.024 5	Deacetylasperulosidic acid methyl ester
5	2.84	$[M-H]^-$	389.108 9	389.108 7	0.883	$C_{16}H_{22}O_{11}$	389.109 2, 209.045 6, 183.066 2, 165.055 6, 147.045 2	Scandoside
6	2.98	$[M-H]^-$	373.114 0	373.113 7	0.639	$C_{16}H_{22}O_{10}$	373.110 4, 211.061 2, 193.050 5, 167.071 2, 149.060 7, 123.045 1	Gardoside*
7	3.13	$[M-H]^-$	391.124 6	391.124 5	1.221	$C_{16}H_{24}O_{11}$	229.071 7, 211.060 9, 185.081 3, 167.071 1	Shanziside*
8	3.32	$[M-H]^-$	403.124 6	403.124 1	1.101	$C_{17}H_{24}O_{11}$	241.072 2, 223.060 7, 193.050 1, 127.040 1, 101.024 6	Gardenosid
9	3.35	$[M-H]^-$	373.114 0	373.113 6	0.478	$C_{16}H_{22}O_{10}$	211.061 8, 193.050 3, 146.060 6, 123.045 0, 167.071 0, 121.056 7	Geniposidic acid*
10	3.38	$[M-H]^-$	403.124 6	403.124 1	-0.561	$C_{17}H_{24}O_{11}$	241.072 2, 223.061 4,139.040 3	Feretoside
11	3.48	$[M-H]^-$	405.140 2	405.139 9	0.503	$C_{17}H_{26}O_{11}$	359.143 5, 197.082 4	Shanziside methyl ester*
12	3.57	$[M-H]^-$	375.129 7	375.129 4	0.715	$C_{16}H_{24}O_{10}$	213.077 4, 169.087 3, 151.076 7	Mussaenosidic acid
13	3.73	$[M-H]^-$	345.155 5	345.155 2	0.775	$C_{16}H_{26}O_8$	113.024 3, 101.024 2, 89.024 3	Jasminoside D
14	3.96	$[M-H]^-$	327.144 9	327.144 7	0.832	$C_{16}H_{24}O_7$	165.092 3	Zataroside B
15	4.04	$[M-H]^-$	353.087 8	353.087 6	1.028	$C_{16}H_{18}O_9$	191.056 4, 179.035 3, 135.045 3	5/3-O-Caffeoyl-quinic acid
16	4.20	$[M-H]^-$	549.182 5	549.181 5	-0.883	$C_{23}H_{34}O_{15}$	225. 077 4, 207. 066 7	Genipin 1-gentiobioside
17	4.87	$[M-H]^-$	387.129 7	387.129 4	0.693	$C_{17}H_{24}O_{10}$	207.066 8, 225.077 4, 433.135 9, 123.045 4,101.024 6	Geniposide*
18	5.34	$[M-H]^-$	345.155 5	345.155 2	0.775	$C_{16}H_{26}O_8$	301.042 5, 165.092 1, 183.102 8, 101.024 4, 89.024 5	Jasminoside B
19	5.49	$[M-H]^-$	353.087 8	353.087 4	0.405	$C_{16}H_{18}O_9$	191.056 6, 173.045 9, 179.065 8, 135.062 5	Chlorogenic acid
20	6.33	$[M-H]^-$	179.035 0	179.034 8	1.832	$C_9H_8O_4$	135.045 4	Caffeic acid
21	6.39	$[M-H]^-$	183.102 7	183.102 3	1.206	$C_{10}H_{16}O_3$	139.113 1, 111.009 0, 137.097 1, 109.065 7, 123.080 5	Jasminodiol
22	6.62	$[M-H]^-$	503.177 0	503.176 9	0.883	$C_{22}H_{32}O_{13}$	223.145 1, 205.087 9, 190.211 3	2-methyl-lerythritol-4-O-(6-O-transsinapoyl)-β-D-glucopyranoside
23	7.01	$[M-H]^-$	359.134 8	359.134 7	1.372	$C_{16}H_{24}O_9$	359.135 1, 197.081 9, 179.070 9	Ixoroside
24	7.44	$[M-H]^-$	429.140 2	429.139 8	0.335	$C_{19}H_{26}O_{11}$	383.133 2, 361.150 6, 239.090 1, 181.087 8	10-acetyl geniposide
25	7.57	$[M-H]^-$	519.150 8	519.150 6	0.614	$C_{25}H_{28}O_{12}$	163.040 1, 145.029 5, 123.045 2, 119.050 2, 149.060 8	6′-O-trans-coumaroyl Geniposidic acid
26	8.24	$[M-H]^-$	551.177 0	551.176 8	0.643	$C_{26}H_{32}O_{13}$	357.119 6, 213.077 5, 193.050 9, 175.040 3	6-O-trans-p-coumaroyl Gardenoside methyl ester
27	9.89	$[M-H]^-$	491.213 4	491.213 3	0.954	$C_{22}H_{36}O_{12}$	167.032 4, 323.101 5, 221.075 2, 161.058 0, 263.081 5	Jasminoside S/H/I
28	10.16	$[M-H]^-$	579.171 9	579.172 1	1.174	$C_{27}H_{32}O_{14}$	205.050 6, 325.093 1, 367.103 7, 385.114 4, 223.061 1, 123.045 1	6′-O-trans-sinapoyl gardoside
29	10.43	$[M-H]^-$	565.192 7	565.192 4	0.574	$C_{27}H_{34}O_{13}$	325.093 6, 295.082 8, 265.072 3, 223.061 8	11-(6-O-trans-sinapoylglucopyranosyl)gardendiol
30	10.79	$[M-H]^-$	609.146 1	609.146 3	1.216	$C_{27}H_{30}O_{16}$	301.035 9, 300.028 1, 271.025 5	Rutin
31	11.60	$[M-H]^-$	465.102 8	465.101 8	0.938	$C_{21}H_{20}O_{12}$	301.035 0, 271.024 5, 255.029 7	Isoquercitrin

32	11.86	[M－H]⁻	593.151 2	593.151 3	1.121	$C_{27}H_{30}O_{15}$	325.092 5, 207.029 6, 93.034 4	Nicotiflorin
33	12.12	[M－H]⁻	755.240 4	755.240 8	1.306	$C_{34}H_{44}O_{19}$	123.076 5, 223.077 4, 205.425 5, 101.218 2, 427.066 5	6′′-O-trans-sinapoylgenipin Gentiobioside
34	12.62	[M－H]⁻	725.229 8	725.230 2	1.201	$C_{33}H_{42}O_{18}$	225.077 3, 193.051 2, 123.045 4	6′′-O-trans-feruloyl genipin Gentiobioside
35	12.69	[M－H]⁻	695.219 3	695.219 2	0.742	$C_{32}H_{40}O_{17}$	663.196 5, 469.136 3, 225.077 3, 145.029 8, 207.066 9	6′′-O-trans-p-coumaroylge nipin gentiobioside
36	13.08	[M－H]⁻	551.213 4	551.213 3	0.742	$C_{27}H_{36}O_{12}$	521.204 1, 367.014 2, 165.092 3	6′-O-trans-sinapoyl Jasminoside L
37	13.34	[M－H]⁻	975.371 5	975.371 0	0.044	$C_{44}H_{64}O_{24}$	651.267 4, 327.160 9, 283.1700, 239.1800	trans-crocin Ⅰ/cis-crocin Ⅰ
38	14.07	[M－H]⁻	593.187 6	593.187 7	1.095	$C_{28}H_{34}O_{14}$	367.105 5, 225.077 2, 223.061 0, 207.065 8, 205.051 2, 123.046 5,	6′-O-sinapoylgeniposide
39	14.64	[M－H]⁻	515.119 5	515.119 1	0.251	$C_{25}H_{24}O_{12}$	353.088 8, 191.056 5, 173.045 8	3,5-Dicaffeoylquinic acid
40	15.51	[M－H]⁻	533.166 4	533.166 3	0.786	$C_{26}H_{30}O_{12}$	145.132 3, 123.130 5, 307.114 8, 225.087 9	6′-O-p-coumaroylgeniposide
41	15.67	[M－H]⁻	659.161 8	659.162 1	1.366	$C_{31}H_{32}O_{16}$	497.131 3, 191.056 4, 161.045 7	3,4-dicaffeovl-5-(3-hydroxy-3-methyl glutaroyl) quinic acid
42	16.44	[M－H]⁻	559.145 7	559.145 5	0.616	$C_{27}H_{28}O_{13}$	397.114 9,173. 045 9	3-caffeoyl-4-sinapoylquinate
43	17.45	[M－H]⁻	535.218 5	535.218 3	0.716	$C_{27}H_{36}O_{11}$	325.092 6, 265.071 5, 223.060 9, 205.050 4	6′-O-trans-sinapoyl Jasminoside A
44	18.57	[M－H]⁻	533.202 8	533.202 5	0.419	$C_{27}H_{34}O_{11}$	205.051 1, 367.104 1, 223.061 3, 165.055 3, 190.027 5	6′-O-trans-sinapoyl Jasminoside C
45	19.58	[M－H]⁻	345.061 6	345.061 4	0.950	$C_{17}H_{14}O_{8}$	315.014 9, 330.038 3, 287.019 9	5,7,3′,4′-tetrahydroxy-6,8-dimethoxy flavone
46	21.75	[M－H]⁻	813.318 7	813.319 2	1.286	$C_{38}H_{54}O_{19}$	651.267 6, 489.213 7, 369.170 9, 327.161 1, 283.170 9, 179.056 4	Crocin II

* represented components compared with reference standards;
